# Flexible, Fully Printable, and Inexpensive Paper-Based Chipless Arabic Alphabet-Based RFID Tags

**DOI:** 10.3390/s22020564

**Published:** 2022-01-12

**Authors:** Jawad Yousaf, Eqab Almajali, Mahmoud El Najjar, Ahmed Amir, Amir Altaf, Manzoor Elahi, Saqer Saleh Alja’afreh, Hatem Rmili

**Affiliations:** 1 Department of Electrical, Computer and Biomedical Engineering, Abu Dhabi University, Abu Dhabi P.O. Box 59911, United Arab Emirates; 1047693@students.adu.ac.ae (M.E.N.); 1061758@studentsaduac.onmicrosoft.com (A.A.); 2 Department of Electrical Engineering, University of Sharjah, Sharjah P.O. Box 27272, United Arab Emirates; ealmajali@sharjah.ac.ae; 3 Department of Electrical and Computer Engineering, Sungkyunkwan University, Suwon 16419, Korea; amiraltaf@skku.edu (A.A.); manzoorelahi19@gmail.com (M.E.); 4 Electrical Engineering Department, Mutah University, Al-Karak 61710, Jordan; eng.saqer-jaa@mutah.edu.jo; 5 Electrical and Computer Engineering Department, Faculty of Engineering, King Abdulaziz University, P.O. Box 80204, Jeddah 21589, Saudi Arabia; hmrmili@kau.edu.sa

**Keywords:** chipless RFID, Arabic alphabets, printable RFID, cheap RFID, paper-based RFID, radar cross-section (RCS), font type, font size

## Abstract

This work presents the design and analysis of newly developed reconfigurable, flexible, inexpensive, optically-controlled, and fully printable chipless Arabic alphabet-based radio frequency identification (RFID) tags. The etching of the metallic copper tag strip is performed on a flexible simple thin paper substrate (*ϵ*_r_ = 2.31) backed by a metallic ground plane. The analysis of investigated tags is performed in CST MWS in the frequency range of 1–12 GHz for the determination of the unique signature resonance characteristics of each tag in terms of its back-scattered horizontal and vertical mono-static radar cross section (RCS). The analysis reflects that each tag has its own unique electromagnetic signature (EMS) due to the changing current distribution of metallic resonator. This EMS of each tag could be used for the robust detection and recognition of all realized 28 Arabic alphabet tags. The study also discusses, for the first time, the effect of the change in font type and size of realized tags on their EMS. The robustness and reliability of the obtained EMS of letter tags is confirmed by comparing the RCS results for selective letter tags using FDTD and MoM numerical methods, which shows very good agreement. The proposed tags could be used for smart internet of things (IoT) and product marketing applications.

## 1. Introduction

The demand for chipless radio frequency identification (RFID) tags has increased in recent times for the accurate recognition of the products and items [1,2,3]. RFID tags are also contributing towards the green world because of their wireless transmission, battery-less structure, lower operating power requirements, energy harvesting, smart sensors design, and usage of natural materials as substrates [4,5,6]. The low-cost fabrication of passive chipless RFID tags makes them a favorite choice for prominent applications such as inventory management in warehouses, cheap cargo, library material and vehicle tracking, access control, document security, healthcare industry, and smart IoT devices [7,8,9,10,11].

The operating mechanism of the passive chipless RFID tags is based on the interaction between the reader system and the reflected waves from the metallic resonant tag. The identification and encoding of the resonant metallic tag is performed based on the captured back-scattered electromagnetic signature (EMS) resonance characteristics of the tag by the reader [12,13,14]. The spectral characteristics of the tag vary with the change in the tag structure and geometry [15,16,17].

Frequency domain chipless RFID tags are classified into two main categories of the alphabet and non-alphabet tags. The non-alphabet resonator tags are available in various shapes and structures such as shorted dipoles [18], curve shape [19], spiral [20], L-shape [13,16], slot-loaded patches [17], stub-loaded structure [21], multiresonator without ground plane [22], and stacked multilayer patches [23], to name a few.

The examples of later category are Latin alphabets [1,6,9,24,25,26], Arabic letters [4,12], and peyote symbols [1,2]. The study of [27] reflects that Latin letter tags could also be used as dipole antennas. The usage of metallic letters for the RFID applications offers advantages of visual identification for the line of sight and radio frequency recognition for non-line-of-sight configurations [1,2,9]. The unique EMS of each alphabet tag depends on its structural configuration (font size, type, spacing, etc.) [1,4,9,25]. The combination of the alphabets results in a word which exhibits its own signature EMS and could be used for the IoT applications, product advertisements, items marketing, inventory management, and documents security by using the metallic product/item name as the RFID tag. This would also reduce the additional associated cost of installation of a separate barcode or non-alphabet-based RFID tag for each product.

**Figure 1:**
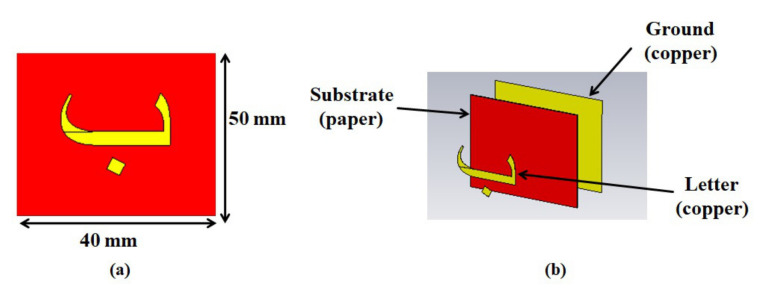
Design of Arabic letter **ب** (baa) tag: (**a**) Tag dimensions; (**b**) Structure of the tag.

This study presents a detailed analysis of the flexible, reconfigurable, cheap, newly designed chipless Arabic letter-based tags. Initially, the tags are designed by etching the Arabic letter of Arial font size of 28 mm on a thin paper substrate (*ϵ*_r_ = 2.31) of 40 × 50 mm which is backed by a copper ground plane, as illustrated in Figure 1 for Arabic **ب** (baa) alphabet. The copper is chosen as the metallic letter tag and ground plane material for all realized 28 Arabic letter tags. The thickness of the metallic letter tag and substrate material is 0.1 mm and 0.5 mm, respectively. The unique signature EMS of each tag is computed in terms of its backscattered monostatic RCS by exciting it with a dual-polarized plane wave in CST MWS in the frequency range of 1 to 12 GHz (details in Section 2). The presented tags constitute a ground plane for improved sensitivity and selectivity of spectral contents and are more flexible due to paper substrate than reported tags in [4,12]. In addition, unlike fixed Arial font size studies of [1,2,4,9,12], this study also presents, for the first time (as per our best knowledge), a detailed analysis of the effect of change in the font type and size of Arabic RFID tags by designing letter tags with Calibri and Times New Roman fonts with font sizes of 28 mm, 20 mm, and 16 mm, respectively. The variations in the resonance frequencies of the tags are analyzed in terms of frequency shift, and an algorithm could be developed for the robust detection and encoding of the investigated tags. Along with analysis of all realized tags using the finite difference time domain (FDTD) technique in CST, the reliability of analyzed tags functions is also verified using the method of moments (MoM) method in Feko (details in Section 5). For IoT application, the name of any sensor (e.g., temperature, location, etc.) or any IoT device in the developed IoT system (e.g., for inventory management, supply chain, vehicle tracking, health, security) could be printed using metallic ink, and thus it can be used as RFID instead of installing a separate dedicated RFID resonator or barcode for that sensor/IoT device.

The organization of the paper is as follows: Section 2 details the working principle for the robust design of the tags encoding system. The discussion about the design procedure and analysis setup of the tags is presented in Section 3. The results and discussion for all 28 designed tags is presented in Section 4. The comparison of selective tags results using FDTD and MoM techniques is discussed in Section 5. Section 6 details the impact of change in font type and size on tags EMS. Lastly, Section 7 concludes the study.

## 2. Working Principle

**Figure 2:**
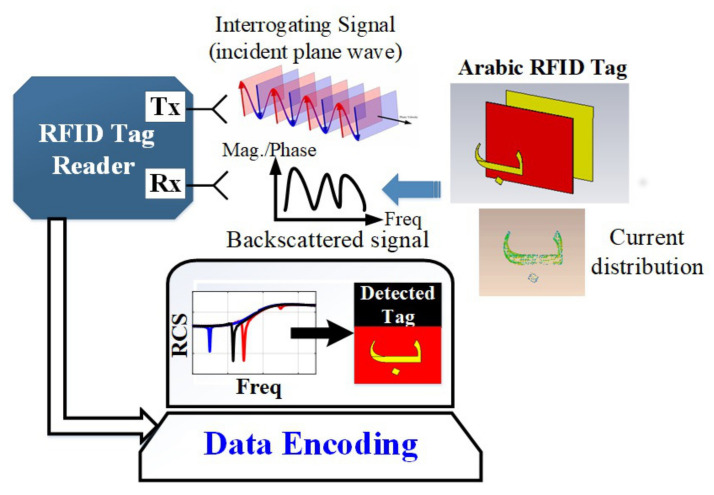
Operation principle of designed chipless Arabic alphabet tags.

In the chipless RFID tags, the antenna at the tag is removed. The detection of the tag is based on the reflected information from the tag which represents its unique resonance characteristics. Figure 2 depicts the operating principle of the designed chipless Arabic letter tags.

The tag is illuminated by an incipient plane wave from the reader dual-polarized transmit (Tx) antenna. It induces the current distribution around the metallic structure of the tag which varies with the change in the font type, size, spacing, and structure (addition of punctuation) of the designed tag. The back-scattered signal is captured by the receiver (Rx) antenna of the reader. The reader analyzes the unique spectral resonance characteristics from the computed radar cross section (RCS) of the received signal. A look-up table can be formed based on the frequency shift coding for each tag ID. The encoding of the tag can be performed based on its signature resonances in a computing software by comparing the obtained results with the assigned frequency shift code of each tag ID. The accuracy of the recorded response by the reader also depends on the isolation of the Tx and Rx antennas.

## 3. Design Process and Analysis Setup

The Arabic letters could be written in a wide range of fonts and sizes. Firstly, we used the Arial font type with a size of 28 mm, owing to its wide popularity, for the realization of all 28 Arabic tag IDs.

**Figure 3:**
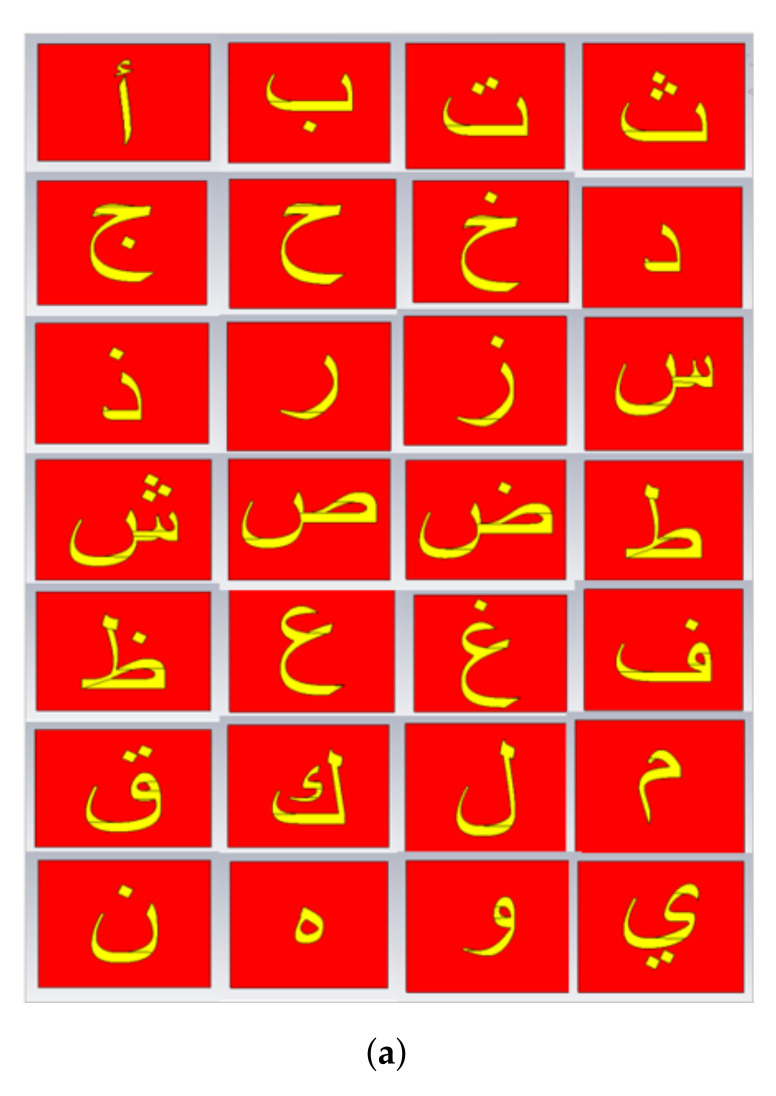

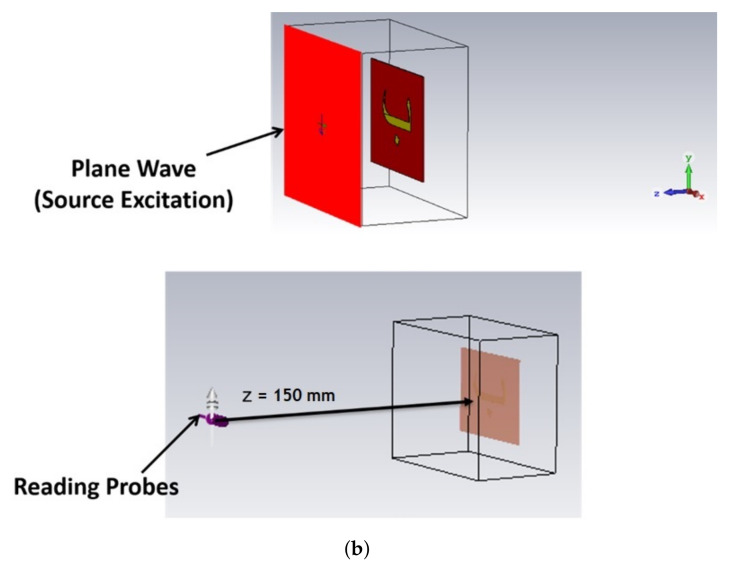
Realized tags and simulation setup: (**a**) All designed Arabic letter tags with Arial font size of 28 mm; (**b**) Tag simulation analysis setup in CST MWS using FDTD method.

The first step in the design of the tag ID is the drawing of the alphabets using 3D computer-aided design software (CAD). We used AutoCad for this purpose and wrote the letters using the text option. After performing the necessary operations for the 3D design of the drawn letter, the letter file is exported to *.sat format. The next step is the full-wave EM analysis of the designed tag. For this purpose, the 3D file of the tag ID is imported to the CST MWS. The exported alphabet is placed at the center of a paper substrate structure having a thickness of 0.5 mm. A ground plane is created at the backside of the substrate. Figure 1 illustrates the layout of the design Arabic alphabet **ب** tag. The copper was used as the metallic material of all designed tags and ground plane. The thickness of the metallic tag shape was 0.1 mm, while 0.5 mm thick paper having a relative permittivity of 2.31 was used as a substrate. Figure 3a depicts all investigated Arabic alphabet tags.

Figure 3b shows the full-wave numerical analysis setup for the designed Arabic letter **ب** tag. The tag was excited with a plane wave as illustrated in Figure 3b. The horizontal and vertical reading probes are placed in the far-field region at a distance of 150 mm (*z*) to capture the reflecting resonance characteristics of each tag in terms of horizontal and vertical RCS. The distance “*z*” satisfies the condition of “*z* ≥ 2*D*/*λ*”, where *D* is the maximum size of the tag and *λ* is the free-space wavelength at the lowest operating frequency. The analysis is performed in the frequency range of 1 to 12 GHz.

## 4. Results and Discussion

**Figure 4:**
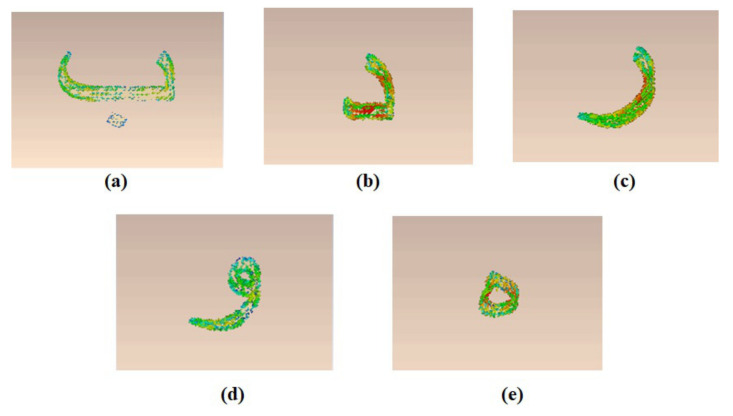
Current distribution of selective designed Arabic tags at their resonance frequencies: (**a**) letter **ب**; (**b**) letter **د**; (**c**) letter **ر**; (**d**) letter **و**; (**e**) letter **ه**.

Figure 4 shows the current distribution of the designed letters **ب** (baa), **د** (dal), **ر** (raa), **و** (waw), and **ه** (ha) tags at the resonance frequency of each tag. The results depict that the change of the tag geometry varies the current distribution of the metallic resonant tag structure. The localized change of the current distribution around the metallic structure of the tag generates the different resonance spectrum of each tag. This unique electromagnetic signature (EMS) could be used for the robust identification and detection of each tag.

**Figure 5:**
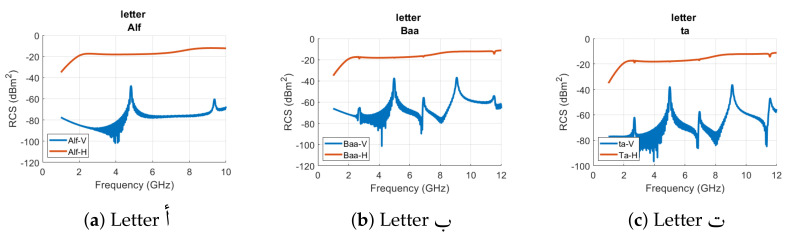

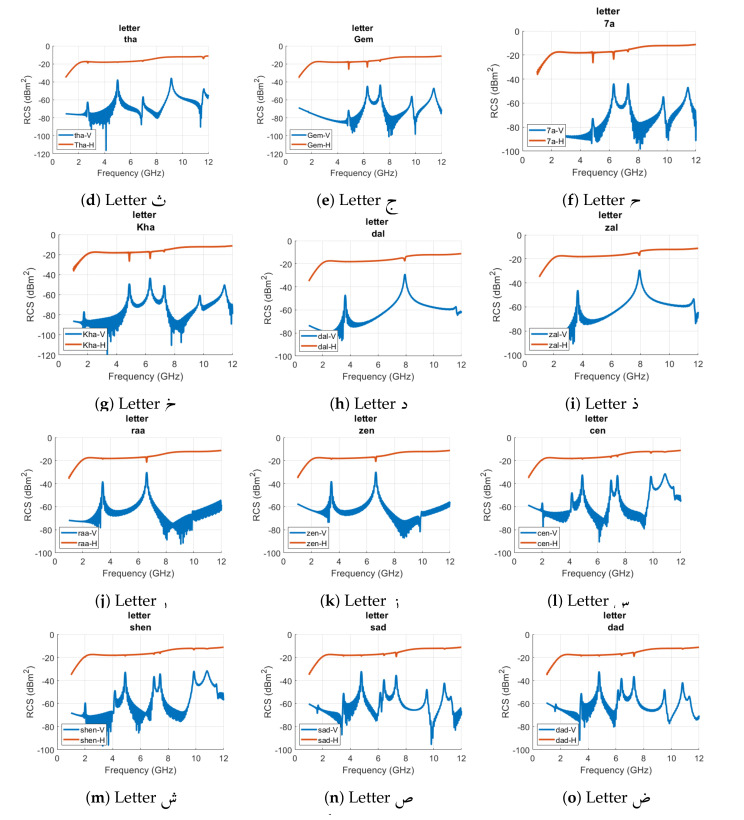
RCS results of Arabic letter tags (**أ**, **ب**, **ت**, **ث**, **ج**, **ح**, **خ**, **د**, **ذ**, **ر**, **ز**, **س**, **ش**, **ص**, **ض**) with Arial font type and font size of 28.

**Figure 6:**
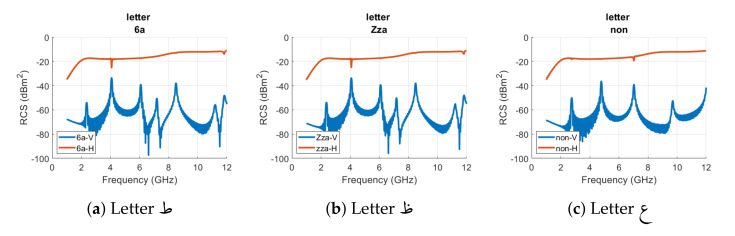

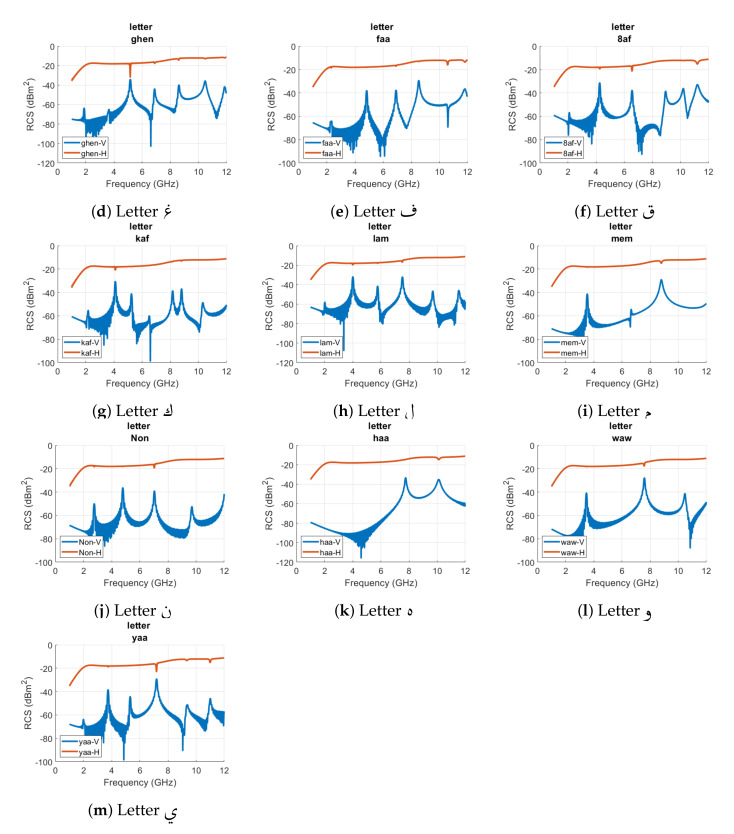
RCS results of Arabic letter tags (**ط**, **ظ**, **ع**, **غ**, **ف**, **ق**, **ك**, **ل**, **م**, **ن**, **ه**, **و**, **ي**) with Arial font type and font size of 28.

Figure 5 and Figure 6 show the horizontal and vertical RCS results of all designed tags of Figure 3a. In Figure 5 and Figure 6, the blue curve represents the vertical (V) polarization RCS while the orange curve depicts the horizontal (H) polarization RCS properties of the analyzed alphabet tag ID, respectively. We can note two distinct resonance peaks for the vertical RCS curve of letter tag alf (**أ**) at 4.8160 GHz and 9.37 GHz, respectively, in Figure 5a. The results of Figure 5b illustrate that the signature resonance frequencies of tag ID **ب** for the vertical polarization are at 2.7380 GHz, 4.9820 GHz, 6.9180 GHz, 9.0850 GHz, and 11.5270 GHz, respectively. The relatively smaller dips in the horizontal polarization RCS of the same tag at the resonance frequencies of 2.705 GHz and 11.538 GHz can be observed in Figure 5b. The comparison of these two tags’ RCS spectrum reflects that both tags exhibit unique signature resonance properties which could be used for their robust detection and identification.

The changes in the resonant characteristics of the remaining tag IDs can be noticed from Figure 5 and Figure 6, respectively. Table 1 summarizes the resonance frequencies in the vertical (cross) and horizontal (co) polarization of all analyzed Arabic alphabet tags. The results show that resonance peaks in the cross-polar RCS are more prominent compared to the dips in the co-polar RCS results of all tags. We can merge the various tags in terms of small differences in the resonance frequencies of those tags. This results in eight groups of tags, i.e., ب, ت, ث as a first group, ج, ح, خ as a second group, د, ذ as a third group, ر, ز as a fourth group, س, ش as a fifth group, ص, ض as a sixth group, ط,ظ as a seventh group, and ع, غ as the eighth group. For all these groups, the variance among the tag geometries of the same group alphabets are in terms of the punctuation (addition of dots).

We can notice from Figure 5b–d and Table 1 that for the aforementioned first group of tags (**ب**, **ت**, **ث**), the variations between the vertical polarization peaks frequencies are not large for the tag IDs **ت** and **ث**. The spectral resonance signature of the tag ID **ب** is different from alphabet tag IDs of **ت** and **ث**. The addition of one additional dot in the alphabet **ث** did not produce a noticeable change in the spectrum waveform for these two tags. The small changes in the tag geometry did not bring any noteworthy variations in the localized current distributions of these tags. The difference occurs in the third or fourth decimal points of resonance frequencies, as depicted in Table 1 for both co- and cross-polar RCS results.

Similar observations can be made for the RCS results of other group tags of **ج**, **ح**, **خ**, **د**, **ذ**, **ر**, **ز**, **س**, **ش**, **ص**, **ض**, **ط**, **ظ**, **ع**, and **غ**. Besides these grouped tags, all other tag IDs differ from each other in terms of spectral contents and thus have a unique signature EMS which provides ease in their identification and detection by the reader.

It is deduced that the reader system must incorporate the sensitivity factors for the robust recognition of designed tags. We designed the robust detection and identification systems for the accurate recognition of the tag ID by comparing the resonance frequencies in both co- and cross-polarization to the fifth decimal point. Additionally, the RCS magnitude at resonance frequencies could be compared for those cases where the tag IDs have the same resonance frequencies, even to the 3rd–5th decimal points, for the accurate identification of the tags. For further distinguishing the electromagnetic responses of the aforementioned eight group tags, the other approaches involve connecting the dots with the tag geometry by an additional thin metallic strip [4] or introducing slots in the tag geometry [25].

## 5. Numerical Verification

**Figure 7:**
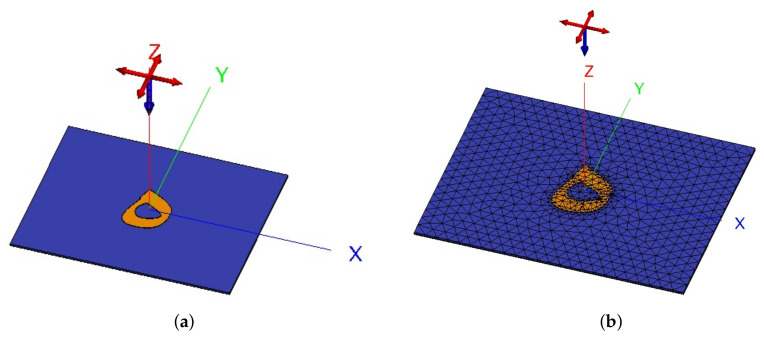
Numerical model of realized letter haa (**ه**) model using MoM technique (**a**) without mesh; (**b**) with mesh.

utf8 The proposed Arabic letter models were rigorously molded using reliable full-wave simulators using two different numerical methods, finite difference time domain (FDTD) in CST and method of moments (MoM) in FEKO. The purpose was to ensure the reliability of the proposed approach. Figure 7 depicts the full-wave analyzed models of letter tag **ه** in FEKO using MoM method. The analysis was performed for the two letter tags **ب** and **ه** to signify the reliability of obtained results using another full-wave EM analysis approach. The numerical simulations settings for both FEKO and CST MWS were the same.

**Figure 8:**
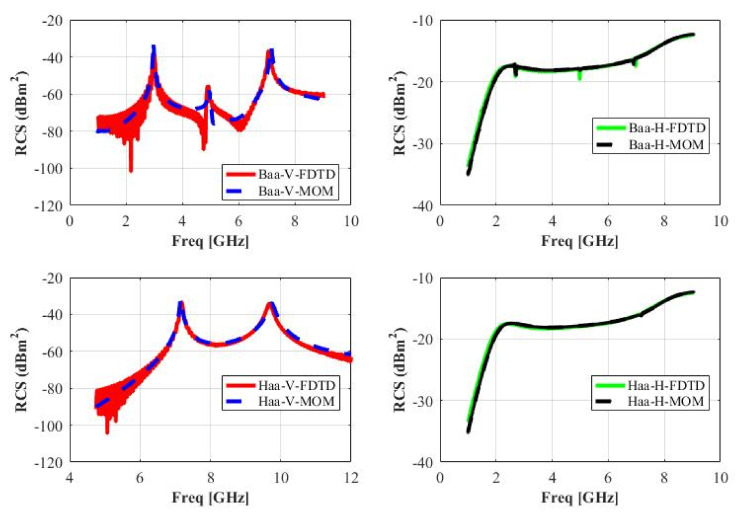
Comparison of RCS characteristics of letters **ب** and **ه** using FDTD and MoM techniques.

Figure 8 illustrates the comparison of obtained RCS waveforms in vertical and horizontal polarization for realized letter tags **ب** and **ه**. It can be noted that obtained waveforms using the MoM approach have very good agreements with the FDTD results for both letter tags in horizontal and vertical polarizations, respectively. The FDTD and MoM technique results match well in terms of wave from characteristics and resonance frequencies of each tag in both polarization. This proves the reliability of the obtained results using the FDTD approach in CST for all realized Arabic alphabet tags.

## 6. Effects of Change in Font Type and Size on EMS

utf8 The impact of the variations in the font size and font type of the realized Arabic alphabet tags to demonstrate the reconfigurable nature of the investigated tags is discussed in this section.

### 6.1. Effect of Change in Font Type

utf8 All 28 Arabic alphabets of Figure 1 are redesigned for Calibri and Times New Roman (TNR) font types. The size of the tag is kept the same, i.e., 28 for the newly designed tags, too. The RCS results of the selective alphabet tag IDs (د, ر, ه, and و) with different font types are discussed here for brevity. The purpose is to demonstrate the impact of writing the letters with various fonts on their EMS.

**Figure 9:**
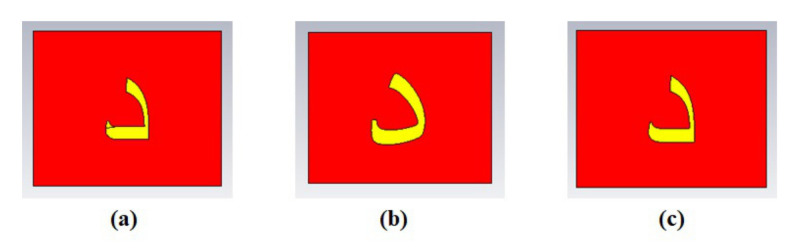
Designed letter **د** tag with various fonts with the same size of 28: (**a**) Arial; (**b**) Calibri; (**c**) Times New Roman (TNR).

**Figure 10:**
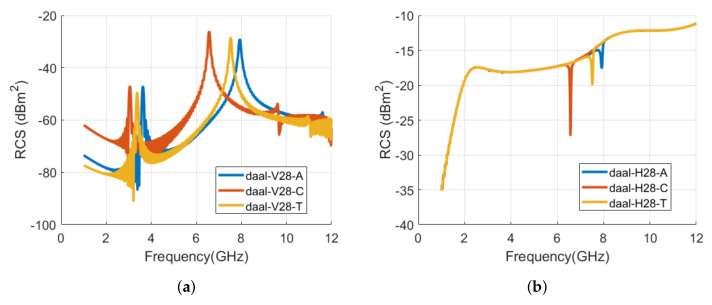
Comparison of RCS results of alphabet **د** tag with various font types (Arial, Calibri, and Times New Roman) with font size of 28: (**a**) Vertical polarization; (**b**) Horizontal polarization.

Figure 9 shows the realized tag IDs of the alphabet **د** with Arial, Calibri, and Times New Roman (TNR) font types in CST MWS. The RCS results for the co- and cross-polarization cases for the analyzed tags of Figure 9 are shown in Figure 10. Table 2 lists the signature resonance frequencies of tag ID **د** for the waveforms of Figure 10.

We notice from the comparison results of Figure 10 that the resonance frequencies of the tags shift backward for the Calibri and TNR fonts when compared with the Arial font type tag. The change in the geometry of the tag for the different font varies its localized current distribution and thus produces the different EMS as illustrated in Figure 10. The first resonance frequency of the Calibri and TNR tags is shifted by −16.1 % (−0.583 GHz) and −7.0 % (−0.253 GHz) as compared to the first resonance frequency of Arial tag in vertical polarization. The frequency shift is computed using Equation (1). In Equation (1), $f_{r}^{org-tag}$ is the resonance frequency of the original tag, i.e., Arial tag resonance frequency here. The $f_{r}^{mod-tag}$ refers to the resonance frequency of the modified tag, i.e., resonance frequency of the Calibri or TNR tag.

(1) $$\Delta f_{r}\left(\%\right)=\frac{f_{r}^{mod-tag}-f_{r}^{org-tag}}{f_{r}^{org-tag}}$$

We can observe that the decrease in resonance frequencies is more for the Calibri font, compared to Times New Roman, when the RCS characteristics of both font tags are compared with spectral EM proprieties of the Arial font-type tag. The larger shift for the Calibri **د** tag is due to the significant variation in its structure, compared to the changes in the tag geometry of the TNR tag when compared with the Arial font tag geometry (see Figure 9). In the case of horizontal frequencies, we noticed that the analysis remains the same but we have fewer variations in the RCS waveform compared to the vertical polarization RCS results.

**Figure 11:**
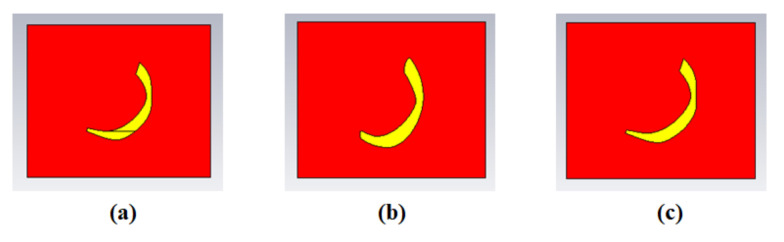
Designed letter **ر** tag with various fonts with the same size of 28: (**a**) Arial; (**b**) Calibri; (**c**) Times New Roman (TNR).

**Figure 12:**
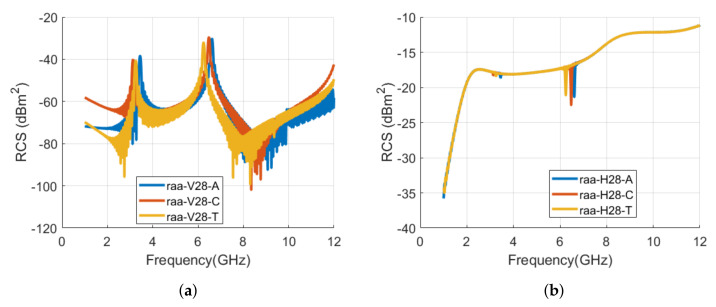
Comparison of RCS results of alphabet **ر** tag with various font types (Arial, Calibri, and Times New Roman) with the font size of 28: (**a**) Vertical polarization; (**b**) Horizontal polarization.

The realized letters tags for the alphabet **ر** with the change in font type are depicted in Figure 11. The vertical and horizontal RCS waveforms for these tags are shown in Figure 12. Similar to tag ID **د**, the resonance frequencies have a backward shift for **ر** tag also when the font type is changed to Calibri and TNR, as shown in Figure 12 and Table 3. The observed frequency shift for tag ID **ر** in vertical polarization is −8.9% and −6.3% for Calibri and TNR tags, respectively. Similar to alphabet **د** tag, the shift for TNR **ر** tag ID is less compared to Calibri **ر** tag ID when compared with the Arial type tag.

**Figure 13:**
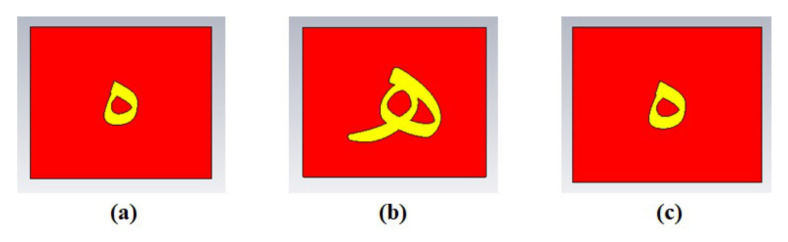
Designed letter **ه** tag with various fonts with the same size of 28: (**a**) Arial; (**b**) Calibri; (**c**) Times New Roman (TNR).

**Figure 14:**
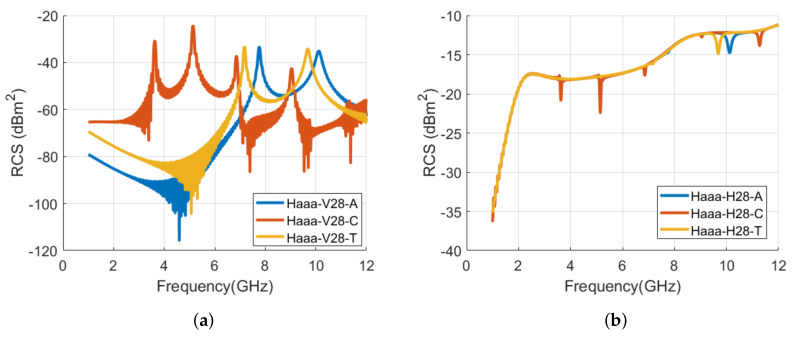
Comparison of RCS results of alphabet **ه** tag with various font types (Arial, Calibri, and Times New Roman) with the font size of 28: (**a**) Vertical polarization; (**b**) Horizontal polarization.

Figure 13 shows the designed tag IDs of alphabet **ه** with Arial, Calibri, and TNR fonts. We can notice that geometry of the tag changes significantly for the Calibri font and that must bring a different EMS of this tag compared to Arial and TNR font-type tag. Figure 14 results confirm that letter **ه** EMS is completely different as compared to the resonance properties of tags with Arial and TNR fonts. The small variations in the tag structure with TNR font bring a small shift in its resonance frequencies (−7.5 %) when compared with the RCS waveform of Arial font-type tag. Table 4 summarizes the signature resonance frequencies for the depicted waveforms of Figure 14.

**Figure 15:**
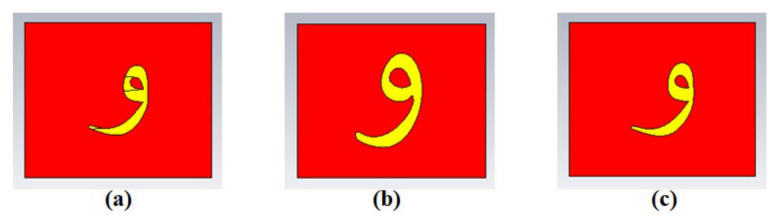
Designed letter و tag with various fonts with the same size of 28: (**a**) Arial; (**b**) Calibri; (**c**) Times New Roman (TNR).

**Figure 16:**
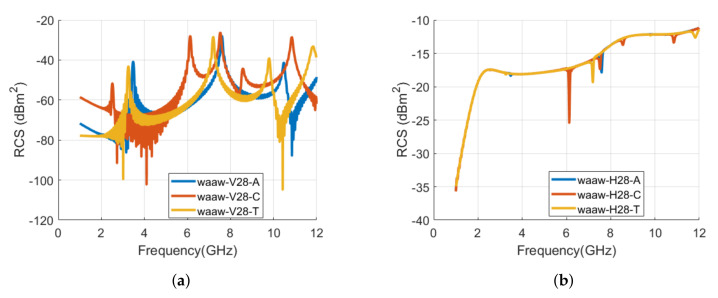
Comparison of RCS results of alphabet **و** tag with various font types (Arial, Calibri, and Times New Roman) with the font size of 28: (**a**) Vertical polarization; (**b**) Horizontal polarization.

The full-wave numerical structures of letter **و** tag are analyzed with different fonts and are depicted in Figure 15. Figure 16 compares the RCS waveforms in vertical and horizontal polarization, while Table 5 lists the corresponding resonance frequencies of the realized tags of Figure 15. The results shows that changing the font to Calibri produces five unique resonance frequencies, while four signature resonating frequencies can be noted for the TNR **و** tag. The corresponding frequency shift in the first resonant frequency of Calibri and TNR tags is –0.968 GHz (−27.9 %) and –0.22 GHz (−6.3 %), respectively, compared to the first resonance frequency of the Arial font-type tag.

Table 6 summarizes the frequency shift in the first resonance frequency for the vertical RCS of all aforementioned presented tags compared to the primary resonance frequency of the Arial type tag. The trend shows that the Calibri font brings a larger shift in the first resonance frequency for all tags compared to TNR tags in comparison with the Arial tag. The reason is less geometrical variation in the tags structure for the Arial and TNR tags compared to the Calibri tags.

### 6.2. Effect of Change in Font Size

In this section, we discuss the comparison between three different font sizes for each tag with three font types (Arial, Calibri, and Times New Roman) to observe the changes in EMS for four Arabic alphabets (**د**, **ر**, **ه**, and **و**). The impact of variations in the font size on resonating frequencies are analyzed by realizing the tags with two additional sizes of 20 mm and 16 mm, respectively.

**Figure 17:**
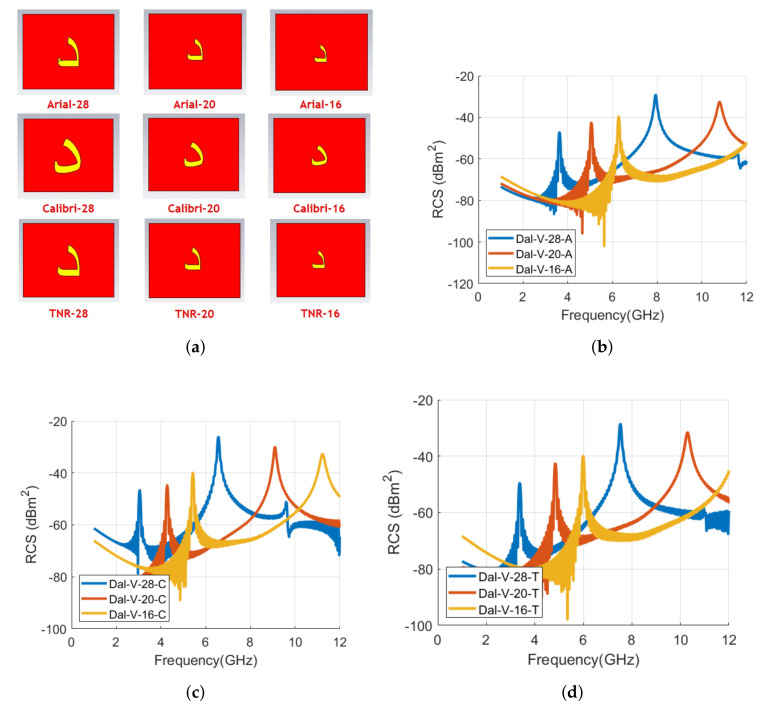
Designed letters and comparison of RCS results of alphabet **د** tag for vertical polarization with various font types and sizes: (**a**) Designed letter **د** tag with various fonts and sizes; (**b**) RCS results of Arial font with 28, 20, and 16 sizes; (**c**) RCS results of Calibri font with 28, 20, and 16 sizes; and (**d**) RCS results of Times New Roman (TNR) font with 28, 20, and 16 sizes.

Figure 17a depicts the analyzed tag IDs of alphabet **د** with three different sizes (28, 20, and 16) for the three different fonts of Arial, Calibri, and Times New Roman, respectively. The substrate and ground plane size was the same as used in the initial analysis of Section 4.

The RCS waveforms for vertical polarization obtained after full-wave EM analysis of Figure 17a tags are shown in Figure 17b–d. The horizontal polarization waveforms are not reported here for brevity. The signature resonating frequencies representing EMS of each tag ID in both horizontal and vertical polarization for all analyzed font types and sizes are shown in Table 7. We note from Figure 17b that for the Arial font type, as the size of the tag reduces, resonating frequencies are shifting towards the higher frequencies. Keeping Arial 28 tag size as a reference, the first resonance frequency is shifted by 39.4% (1.43 GHz) and 73.1% (2.651 GHz) when the tag size is reduced to 20 and 16, respectively. The frequency shift is calculated using Equation (1). For the Calibri font type, the % increase in the first resonance frequency is 40.4% and 78.0%, respectively, with the tag IDs of sizes 20 and 16, respectively. The analysis for the TNR tag sizes reflects that TNR-20 increases the first resonance frequency by 43.7% while TNR-16 brings a positive increase of 77.5% in the corresponding first resonance frequency of the TNR-28 tag, i.e., 3.376 GHz. A similar trend of shifting of the second resonance frequency towards higher frequencies can be noticed for the tags of different sizes for Calibri and TNR fonts (see Figure 17c,d).

It is important to mention here that the change of the font size did not bring a change in the number of resonance frequencies or the spectral shape of the tag. We can notice from Figure 17b that the Arial-28 size waveform has two resonant frequencies, i.e., 3.629 GHz and 7.93 GHz. The reduction of tag size to 20 shifts the resonant frequencies to higher levels (5.059 GHz and 10.79 GHz) with no change in the number of resonant frequencies. The further decrease in size to 16 results in the same behavior. However, the second resonate frequency for the tag size of 16 is not visible in Figure 17b, as its beyond the analyzed frequency limit of 12 GHz. This is why Table 7 lists only one signature resonant frequency for Arial-16 size tag ID, as the existing second resonance frequency (which is denoted by * in Table 7) is out of the analyzed frequency range.

**Figure 18:**
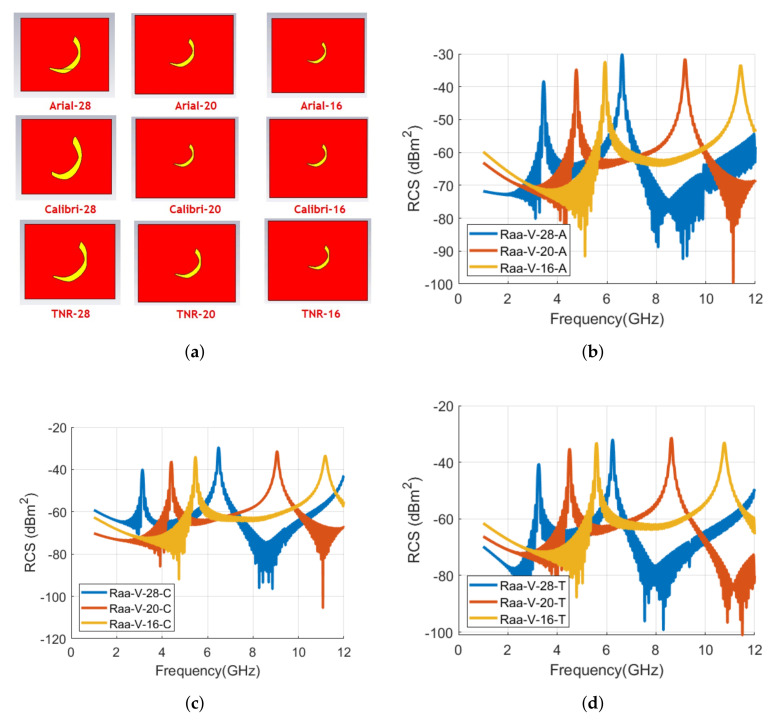
Designed letters and comparison of RCS results of alphabet **ر** tag for vertical polarization with various font types and sizes: (**a**) Designed letter **ر** tag with various fonts and sizes; (**b**) RCS results of Arial font with 28, 20, and 16 sizes; (**c**) RCS results of Calibri font with 28, 20, and 16 sizes; and (**d**) RCS results of Times New Roman (TNR) font with 28, 20, and 16 sizes.

Figure 18 shows the comparison of the vertical polarization RCS results for the letter tag ر for the analyzed three different font types and sizes. The corresponding signature resonance frequencies to these waveforms are depicted in Table 8. The alphabet tag ر has two signature resonant frequencies with Arial-28 tag. It can be observed from Figure 18 and Table 8 that, similar to letter tag **د** results, the resonance frequencies are transposed to a higher range with the decrease in the tag size. The observed frequency shifts in the primary resonant frequency for the letter tag **ر** in vertical polarization are 1.0 % and 71.9 % for Arial font, 38.4 % and 74.4 % for Calibri font, and 38.7 % and 72.2 % for TNR font, respectively, for the reference 28 font size of each font type.

**Figure 19:**
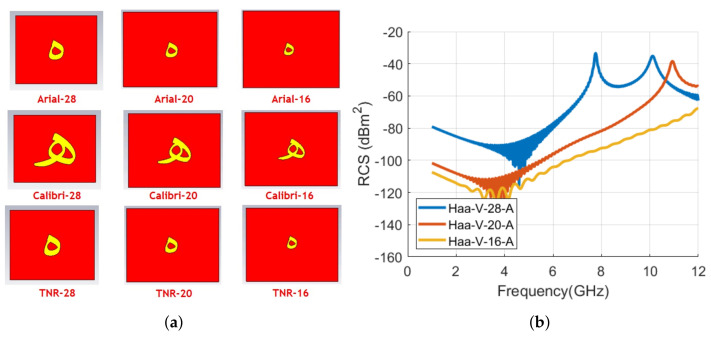

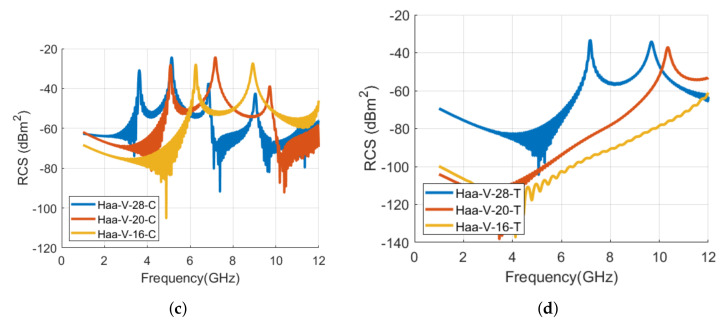
Designed letter and comparison of RCS results of alphabet **ه** tag for vertical polarization with various font types and sizes: **(a**) Designed letter **ه** tag with various fonts and sizes; (**b**) Arial font with 28, 20, and 16 sizes; (**c**) Calibri font with 28, 20, and 16 sizes; and (**d**) Times New Roman (TNR) font with 28, 20, and 16 sizes.

The comparison of the RCS vertical polarization spectral components for the alphabet tag **ه** with the change in tag size for the realized font types is depicted in Figure 19. Table 9 illustrates the obtained resonant frequencies from the waveforms of Figure 19.

**Figure 20:**
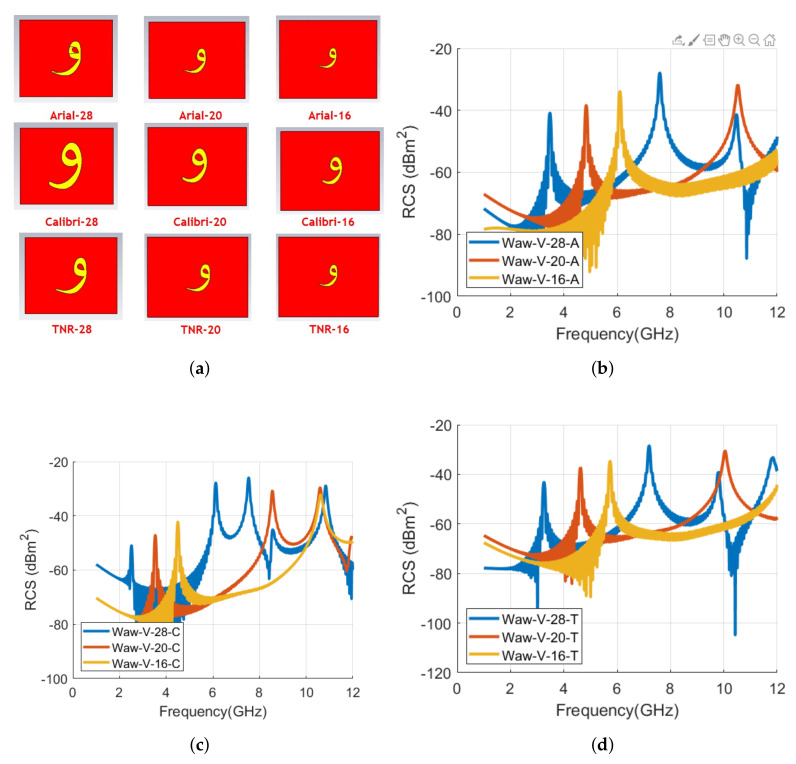
Designed letters and comparison of RCS results of alphabet **و** tag for vertical polarization with various font types and sizes: (**d**) Design letter **و** tag with different fonts and sizes; (**b**) Arial font with 28, 20, and 16 sizes; (**c**) Calibri font with 28, 20, and 16 sizes; and (**d**) Times New Roman (TNR) font with 28, 20, and 16 sizes.

Figure 20 depicts the variations in the RCS properties for the letter tag **و**. The signature resonance frequencies of Figure 20 waveforms are listed in Table 10. We can note the similar trend of transformation of resonance frequenters to higher frequencies when the tag size is contracted for the letter tags **ه** and **و** respectively. Table 11 summarizes the frequency shift (Δ*f*) in vertical polarization for the investigated Arial, Calibri, and TNR tags with the variations in their font sizes. It can be noted from Table 11 that the reduction of the tag size to 16 mm brings a positive shift of more than 70% in the primary resonance frequency of all the font type tags, compared to the reference 28 size tag.

The analysis of the changing font type and font size confirms that the metallic tag structure for different font types and sizes has a significant impact on the EMS of the tag. The variation of the font type from Arial to Calibri to Times New Roman with fixed font size mainly shifts the resonance frequencies to the lower range. In addition, the tag geometry has a noticeable change for the Calibri font when compared with the Arial or Times New Roman font structures. The significant difference of tag structure in Calibri font changes the resonance spectrum (# of resonance frequencies) of the tag, along with shifting of the resonance frequencies. On the contrary, irrespective of the tag font type, the decrease of the font size only shifts the resonant frequencies to the higher range, as the smaller electrical tag resonates at higher frequencies. This did not bring any change in the spectral components of the tags, i.e., the number of resonant frequencies.

The analysis reflects that both font type and font size have a noticeable impact on the EMS of the tag. For the marketing and advertisement applications of the products, we can print the same product/item name with various font types or font sizes for different companies. This will produce the unique identity of each item, thus easily differentiating the same product name of different companies, because of the distinct EMS of the tag with a change in font types and sizes. This will also provide the security features to the designed tags due to unique spectrum components of each tag ID. Furthermore, the optical control (visual recognition) and detection of all resonances without any ambiguity offer additional advantages to the proposed Arabic letter tag IDs as compared to the reported just resonant metallic shape tags in [13,16,17,18,19,20,21,22,23].

Table 12 compares the proposed design of chipless Arabic alphabet-based RFID tags with reported alphabet-based tags in literature. The reported studies of [2,6,9,24,25] concern the Latin letter tags with various metallic materials and substrate types. Among the reported studies of [1,2,6,9,24,25], only [9] presented the impact of the structural modifications on the tags for the Latin alphabet letter tags. This work has developed the more flexible paper-based Arabic tags with higher spectral sensitivity as compared to the reported design in [4,12]. Furthermore, the proposed study has demonstrated, for the first time, the impact of the structural modifications in terms of various font types (Arial, Calibri, and Times New Roman) and sizes (16, 20, and 28) on the Arabic tags’ electromagnetic structures.

## 7. Conclusions

This study has reported the design of newly developed, reconfigurable, flexible, cheap, optically controllable, and fully printable Arabic letter-based chipless RFID tags for product advertisement, document security, and IoT applications. The proposed 28 Arabic alphabet-based tags are realized by etching the metallic copper letter tag on a thin flexible paper substrate in CST MWS in the frequency range of 1 to 12 GHz. The RCS analysis of designed tags deduced that each tag has a unique electromagnetic signature (EMS) which could be used for the identification and detection of each tag. The conducted study of the effect of the change in the geometrical structure of the tags in terms of changing font types (Arial–Calibri–Times New Roman) and font sizes (28–20–16) reflects that changing font types mainly shifts the resonance frequencies of the tags to lower level and vice versa for the decrease in the font size from 28 to 16. The robustness of the EMS of the realized tags is confirmed with the comparison of FDTD results of tags with MoM results. The frequency shift-based algorithm could be developed for the robust encoding and detection of the developed Arabic letter tags.

## Figures and Tables

**Table 1:** Co- and cross-polar resonate frequencies of designed Arabic tag IDs with Arial font type of size 28 (waveforms in Figure 5 and Figure 6).

Tag	Vertical Polarization	Horizontal Polarization
Letter **أ**	4.816–9.37	――-
Letter **ب**	2.738–4.982–6.918–9.085–11.527	2.705–11.538
Letter **ت**	2.694–5.015–6.962–9.096–11.571	2.705–11.56
Letter **ث**	2.694–5.004–6.962–9.14–11.637	2.705–11.615
Letter **ج**	4.839–6.291–7.281–9.701–11.395	4.85–6.28–7.27
Letter ح	4.861–6.313–7.303–9.745–11.439	4.872–6.302–7.292
Letter **خ**	1.748–4.883–6.313–7.292–9.745–11.439	4.784–4.883–6.313–7.2841
Letter د	3.629–7.93–11.604	7.919
Letter **ذ**	3.662–7.941–11.692	7.919
Letter **ر**	3.442–6.61	3.442–6.61
Letter **ز**	3.453–6.654	3.453–6.654
Letter **س**	4.157–4.905–6.984–7.435–9.855–10.867	4.905–7.435–9.844
Letter **ش**	2.023–4.168–4.905–6.984–7.424–9.866–10.801	4.905–7.413–9.855
Letter **ص**	1.671–3.519–4.795–6.159– 6.423–7.303–9.514–10.779	3.508–6.434–7.314
Letter **ض**	1.682–3.508–4.795–6.17–6.412–7.303–9.514–10.812–11.296	3.497–6.412–7.314
Letter **ط**	2.364–4.08–6.093–6.445–7.193–8.513–11.208–11.835	3.981–4.08
Letter **ظ**	2.375–4.08–6.093–7.193–8.502–11.219–11.835	3.981–4.091
Letter **ع**	1.88–3.585–5.169–6.885–8.612–10.449–11.813	5.169–6.874–8.601
Letter **غ**	1.88–3.64–5.158–6.896–8.623–10.482–11.868	5.158–6.896–8.601
Letter **ف**	2.331–4.828–6.918–8.535–11.824	10.592–11.813
Letter **ق**	2.1–4.256–6.566–8.953–10.207–11.197	4.256–6.566–11.197
Letter **ك**	2.166–4.102–5.246–6.511– 8.172–8.81–10.317	4.102
Letter **ل**	2.012–4.014–5.763–7.534–9.69–11.538	4.014–7.523
Letter **م**	3.53–6.478–8.81	8.799
Letter **ن**	2.76–4.795–7.039–9.701	2.76–7.028
Letter **ه**	7.754–10.108	10.119
Letter **و**	3.475–7.6–10.482	7.589
Letter **ي**	1.99–3.739–5.312–7.193–9.382–11.01	3.75–7.182–9.338–10.999

**Table 2:** Signature resonance frequencies for letter **د** tag with various font types (Arial, Calibri, and Times New Roman) with the font size of 28.

Tag د	Vertical Polarization	Horizontal Polarization
Arial	3.629–7.93–11.604	3.629–7.93
Calibri	3.046–6.566–9.613	3.046–6.566
TNR	3.376–7.523	3.376–7.523

**Table 3:** Signature resonance frequencies for letter **ر** tag with various font types (Arial, Calibri, and Times New Roman) with the font size of 28.

Tag ر	Vertical Polarization	Horizontal Polarization
Arial	3.442–6.61	3.442–6.61
Calibri	3.134–6.489	3.134–6.489
TNR	3.244–6.247	3.244–6.247

**Table 4:** Signature resonance frequencies for tag letter **ه** with various font types (Arial, Calibri and Times New Roman) with the font size of 28.

Tag ه	Vertical Polarization	Horizontal Polarization
Arial	7.754–10.108	10.108
Calibri	3.629–5.136–6.852–9.041	3.629–5.136–6.852–9.041–11.252
TNR	7.171–9.679	9.679

**Table 5:** Signature resonance frequencies for letter **و** tag with various font types (Arial, Calibri, and Times New Roman) with the font size of 28.

Tag **و**	Vertical Polarization	Horizontal Polarization
Arial	3.475–7.6–10.482	3.475–7.6
Calibri	2.507–6.126–7.534–8.568–10.834	6.126–7.534–8.568–10.834
TNR	3.255–7.193–9.789–11.846	3.255–7.193–11.846

**Table 6:** Change in resonance frequency (Δ*f*) in % in vertical polarization for analyzed tags for changing font types with reference to Arial font type.

Font Type	Δ*f* (%)			
	**Tag د**	**Tag ر**	**Tag ه**	**Tag و**
Calibri	−16.1	−8.9	−53.2	−27.9
TNR	−7.0	−6.3	−7.5	−6.3

**Table 7:** Signature resonance frequencies for letter tag **د** with various font types and sizes (* represents resonant frequency out of analyzed frequency range).

	Vertical Polarization	Horizontal Polarization
Arial-28	3.629–7.93	7.93
Arial-20	5.059–10.79	10.79
Arial-16	6.28–*	—
Calibri-28	3.046–6.566–9.613	6.566
Calibri-20	4.278–9.096–*	9.096
Calibri-16	5.422–11.23–*	11.23
TNR-28	3.376–7.523	7.523
TNR-20	4.85–10.295	10.295
TNR-16	5.994–*	—

**Table 8:** Signature resonance frequencies for letter tag **ر** with various font types and sizes.

	Vertical Polarization	Horizontal Polarization
Arial-28	3.442–6.61	3.442–6.61
Arial-20	4.762–9.162	4.762–9.162
Arial-16	5.917–11.406	5.917–11.406
Calibri-28	3.134–6.489	3.134–6.489
Calibri-20	4.339–9.063	4.339–9.063
Calibri-16	5.466–11.175	5.466–11.175
TNR-28	3.244–6.247	3.244–6.247
TNR-20	4.498–6.247	4.498–6.247
TNR-16	5.587–10.768	5.587–10.768

**Table 9:** Signature resonance frequencies for letter tag **ه** with various font types and sizes (* represents resonant frequency out of analyzed frequency range).

	Vertical Polarization	Horizontal Polarization
Arial-28	7.754–10.108	10.108
Arial-20	10.933–*	–
Arial-16	*–*	–
Calibri-28	3.629–5.136–6.852–9.041	3.629–5.136–6.85–9.041–11.252
Calibri-20	5.081–7.171–9.041–*	5.081–7.171–9.712
Calibri-16	6.258–9.679–*–*	6.258–8.92
TNR-28	7.171–9.679	9.679
TNR-20	10.35–*	–
TNR-16	–*–*	–

**Table 10:** Signature resonance frequencies for letter tag **و** with various font types and sizes (* represents resonant frequency out of analyzed frequency range).

	Vertical Polarization	Horizontal Polarization
Arial-28	3.475–7.6–10.482	3.475–7.6
Arial-20	4.839–10.526–*	4.839–10.526
Arial-16	6.104–*–*	6.104
Calibri-28	2.507–6.126–7.534–8.568–10.834	6.126–7.534–8.568–10.834
Calibri-20	3.53–8.557–10.603–*	8.557–10.603
Calibri-16	4.498–10.614–*–*	10.614
TNR-28	3.255–7.193–9.789–11.846	3.255–7.193–11.846
TNR-20	4.619–10.042–*–*	4.619–10.042
TNR-16	5.73–*–*–*	5.73

**Table 11:** Change in resonance frequency (Δ*f*) in % in vertical polarization for analyzed tags for the changing font size with reference to Arial-28, Calibri-28, and Times New Roman (TNR)-28 tags.

Font Size	Δ*f* (%)			
	**Tag د**	**Tag ر**	**Tag ه**	**Tag و**
Arial-20	39.4	1.0	41.0	39.3
Arial-16	73.1	71.9	-	75.7
Calibri-20	40.4	38.4	40.0	40.8
Calibri-16	78.0	74.4	72.4	79.4
TNR-20	43.7	38.7	44.3	41.9
TNR-16	77.5	72.2	-	76.0

**Table 12:** Comparison of the proposed design of printable chipless RFID Arabic letter tags design with legacy designs.

Ref.	Design Type	Substrate	Metallic Material	Tag Ground Plane	Font Type (Height in mm)	Freq. (GHz)	No. of Resonators per Tag	Size (cm^2^)	RCS Analysis	Analysis of Structural Modifications
[6]	Single letter Latin alphabet	Kapton	Copper	No	Arial (24)	1–10	1	3.7 × 3.7	Yes	No
[24]	Single letter Latin alphabet	Kapton	PEC	No	Arial (48)	1–10	1	4.8 × 4.5	No	No
[1,2]	Peyote symbols of five letters	FR4 and Mylar Polyester Film	Al	No	Blocks building	57–64	5	5 × 1	No	No
[25]	Latin alphabets letters and words with slots	–	–	No	Calibri (53)	1–10	3	5.3 × 9	Yes	No
[9]	Latin alphabet letters and three letters words	TLX-8	Copper	Yes	Arial, Corbel, Times New Roman (15–16.4)	6–13	3	4 × 2	Yes	Yes
[12]	Single letter Arabic Alphabets	Kapton	PEC	No	Arial (24)	1-10	1	3.7 × 3.7	No	No
[4]	Single letter Arabic alphabets	Kapton	Copper	No	Arial with dots (24)	2–8	1	3.7 × 3.7	Yes	No
This work	Arabic alphabet letters	Paper	Copper	Yes	Arial, Calibri, Times New Roman (16, 20, 28)	1–12	1	4 × 5	Yes	Yes

## Data Availability

The data presented in this study are available on request from the corresponding author.
